# Error in Byline

**DOI:** 10.1001/jamanetworkopen.2026.31421

**Published:** 2026-08-07

In the Original Investigation titled “Chronic Conditions and Mortality in Medicare Beneficiaries Before and After the COVID-19 Pandemic,”^1^ published June 12, 2026, there was an error in Anne M. Butler’s name in the byline. This article has been corrected.^1^
